# Physician Characteristics Associated With Ordering 4 Low-Value Screening Tests in Primary Care

**DOI:** 10.1001/jamanetworkopen.2018.3506

**Published:** 2018-10-12

**Authors:** Zachary Bouck, Jacob Ferguson, Noah M. Ivers, Eve A. Kerr, Kaveh G. Shojania, Min Kim, Peter Cram, Ciara Pendrith, Graham C. Mecredy, Richard H. Glazier, Joshua Tepper, Peter C. Austin, Danielle Martin, Wendy Levinson, R. Sacha Bhatia

**Affiliations:** 1 Institute for Health Systems Solutions and Virtual Care, Women’s College Hospital, Toronto, Ontario, Canada; 2 currently a student at Schulich School of Medicine and Dentistry, University of Western Ontario, London, Ontario, Canada; 3 Institute for Clinical Evaluative Sciences, Toronto, Ontario, Canada; 4 Institute for Health Policy, Management and Evaluation, University of Toronto, Toronto, Ontario, Canada; 5 Center for Clinical Management, Department of Veterans Affairs Ann Arbor Healthcare System, Ann Arbor, Michigan; 6 Department of Internal Medicine and Institute for Healthcare Policy and Innovation, University of Michigan, Ann Arbor; 7 Department of Internal Medicine and Institute for Healthcare Policy and Innovation, University of Michigan, Ann Arbor; 8 Department of Medicine, University of Toronto, Toronto, Ontario, Canada; 9 Division of General Internal Medicine and Geriatrics, Sinai Health System and Health Network, Toronto, Ontario, Canada; 10 Cumming School of Medicine, University of Calgary, Calgary, Alberta, Canada; 11 Li Ka Shing Knowledge Institute, St Michael’s Hospital, Toronto, Ontario, Canada; 12 Department of Family and Community Medicine, University of Toronto, Toronto, Ontario, Canada; zoi18016213 **Additional Contributions:** Andrew J. Calzavara, MSc, Institute for Clinical Evaluative Sciences, Toronto, Ontario, Canada, assisted with the final statistical analysis. No compensation was received.

## Abstract

**Importance:**

Efforts to reduce low-value tests and treatments in primary care are often ineffective. These efforts typically target physicians broadly, most of whom order low-value care infrequently.

**Objectives:**

To measure physician-level use rates of 4 low-value screening tests in primary care to investigate the presence and characteristics of primary care physicians who frequently order low-value care.

**Design, Setting, and Participants:**

A retrospective cohort study was conducted using administrative health care claims collected between April 1, 2012, and March 31, 2016, in Ontario, Canada. This study measured use of 4 low-value screening tests—repeated dual-energy x-ray absorptiometry (DXA) scans, electrocardiograms (ECGs), Papanicolaou (Pap) tests, and chest radiographs (CXRs)—among low-risk outpatients rostered to a common cohort of primary care physicians.

**Exposures:**

Physician sex, years since medical school graduation, and primary care model.

**Main Outcomes and Measures:**

This study measured the number of tests to which a given physician ranked in the top quintile by ordering rate. The resulting cross-test score (range, 0-4) reflects a physician’s propensity to order low-value care across screening tests. Physicians were then dichotomized into infrequent or isolated frequent users (score, 0 or 1, respectively) or generalized frequent users for 2 or more tests (score, ≥2).

**Results:**

The final sample consisted of 2394 primary care physicians (mean [SD] age, 51.3 [10.0] years; 50.2% female), who were predominantly Canadian medical school graduates (1701 [71.1%]), far removed from medical school graduation (median, 25.3 years; interquartile range, 17.3-32.3 years), and reimbursed via fee-for-service in a family health group (1130 [47.2%]). They ordered 302 509 low-value screening tests (74 167 DXA scans, 179 855 ECGs, 19 906 Pap tests, and 28 581 CXRs) after 3 428 557 ordering opportunities. Within the cohort, generalized frequent users represented 18.4% (441 of 2394) of physicians but ordered 39.2% (118 665 of 302 509) of all low-value screening tests. Physicians who were male (odds ratio, 1.29; 95% CI, 1.01-1.64), further removed from medical school graduation (odds ratio, 1.03; 95% CI, 1.02-1.04), or in an enhanced fee-for-service payment model (family health group) vs a capitated payment model (family health team) (odds ratio, 2.04; 95% CI, 1.42-2.94) had increased odds of being generalized frequent users.

**Conclusions and Relevance:**

This study identified a group of primary care physicians who frequently ordered low-value screening tests. Tailoring future interventions to these generalized frequent users might be an effective approach to reducing low-value care.

## Key Points

**Question:**

Do physicians who order a high frequency of 1 low-value screening test also order a high frequency of other low-value screening tests?

**Findings:**

In this cohort study of 2394 primary care physicians, 18.4% of the physicians were in the top ordering quintile of at least 2 of 4 low-value screening tests. These physicians ordered 39.2% of all low-value screening tests.

**Meaning:**

The study findings suggest that efforts to reduce low-value care should consider strategies that focus on physicians who order a high frequency of low-value care.

## Teaser

This cohort study measures physician-level use rates of 4 low-value screening tests in primary care—repeated dual-energy x-ray absorptiometry (DXA) scans, electrocardiograms (ECGs), Papanicolaou (Pap) tests, and chest radiographs—to investigate the characteristics of primary care physicians who frequently order low-value care.

## Introduction

The Choosing Wisely (CW) campaign, present in more than 20 countries worldwide, is dedicated to reducing the frequency of low-value care,^1,2,3,4^ which represents little to no patient benefit or comparatively greater risk of harm.^5,6,7^ While the CW campaign has been successful at identifying what constitutes low-value care and raising awareness among members of the medical community and general public,^2,8,9^ the results of the campaign on reducing low-value care outside of focused local efforts have been underwhelming.^3,9,10,11^

To achieve meaningful reductions in low-value care, Kerr and colleagues^9^ recommend that the CW campaign focus on identifying high-priority clinical targets and developing innovative, theory-based implementation and evaluation strategies. To this end, prior studies^7,12,13,14,15,16,17,18,19,20^ have identified several tests, treatments, or procedures labeled as low value by 1 or more CW recommendations that are commonly used in clinical practice with substantial ordering variation among health care professionals. While previous investigations have described ordering rates of individual tests, there has been little research on understanding individual physician ordering patterns and, in particular, whether physicians who frequently order 1 low-value test are more likely to order other low-value tests. Measuring physician-level ordering frequency of low-value care across several CW recommendations may assist in the development of targeted interventions that are more physician centric and less test centric.

The present study aimed to investigate whether physician-level ordering rates of different low-value screening tests are dependent. We hypothesized that physicians who frequently order 1 type of low-value test would be more likely to order other low-value tests with high frequency, thereby exhibiting a general pattern of overuse. We then identified characteristics associated with frequent physician-level ordering across tests.

## Methods

### Study Design and Data Sources

This study followed the Strengthening the Reporting of Observational Studies in Epidemiology (STROBE) reporting guideline. We conducted a retrospective cohort study in Ontario, Canada, using population-based administrative health care databases held at the Institute for Clinical Evaluative Sciences (ICES).^21^ These databases include the following: (1) the Registered Persons Database, which contains demographic information on all Ontario residents eligible for the Ontario Health Insurance Plan; (2) the Ontario Health Insurance Plan database, which includes all billing claims made by physicians to the Ontario Ministry of Health and Long-Term Care; (3) Client Agency Program Enrolment tables, which can identify patients rostered to primary care physicians; and (4) the ICES Physician Database, which contains demographic information on all physicians in Ontario. These data sets were linked using unique encoded identifiers and analyzed at the ICES. Use of data in this project was authorized under §45 of Ontario’s Personal Health Information Protection Act, which does not require review by a research ethics board.

We measured use of 4 screening tests identified as low value in the following clinical scenarios by CW Canada recommendations: (1) repeated dual-energy x-ray absorptiometry (DXA) scans for patients with a prior DXA scan in the past 2 years,^22,23^ (2) electrocardiograms (ECGs) for patients at low risk for cardiovascular disease,^24,25^ (3) Papanicolaou (Pap) tests for women younger than 21 years or older than 69 years,^25^ and (4) chest radiographs (CXRs) for patients at low risk for cardiopulmonary disease.^25^ Previous studies^7,14,15^ of administrative data have demonstrated substantial overuse of these services and/or extensive gaps in performance among groups of physicians. Moreover, we chose these recommendations because they are clinical scenarios routinely encountered in primary care, often as part of an annual health examination (AHE).^22,23,24,25^

Our methods for measuring these low-value care services were adapted from previously published studies^7,14,15^ that similarly measured the frequency of these tests using administrative data at the ICES. These prior studies predominantly measured use among physician billing groups,^7,14,15^ and methods were modified to enable physician-level measurement.^26,27^ eTable 1 in the Supplement summarizes how each recommendation-based cohort (denominator) and test (numerator) were defined.^7,14,15^

### Cohort Creation

We first identified test-specific cohorts composed of health care services considered to be clinical opportunities to order that low-value screening test. Opportunities to order an ECG, a Pap test, or a CXR were defined as AHEs occurring between April 1, 2012, and March 31, 2016, with a primary care physician and an outpatient at low risk for either cardiovascular disease, cervical cancer, or cardiopulmonary disease, respectively.^7,14,15^ Alternatively, an opportunity to order a repeated DXA was defined as a prior DXA scan between April 1, 2012, and March 31, 2014, for a patient 40 years or older.^7^ A shorter accrual window relative to the AHE-based measures was necessary to allow a 2-year observation window for subsequent testing.

For all 3 AHE-based cohorts, the physician responsible for conducting the examination was identified.^14,15^ For the ECG and CXR cohorts, only AHEs with adult patients (18 years or older) were eligible. For the Pap test cohort, only AHEs with female patients between 13 and 20 years old or older than 69 years were included.^7,25^ If a patient had multiple DXA scans within the accrual window, the first DXA was chosen as the index scan and attributed to the patient’s physician at the time of the scan. We excluded any opportunities involving patients with an invalid health card number, those older than 105 years, or individuals with incomplete or missing demographic data (age, sex, or postal code).^7,14,15^ Individual patients could contribute multiple opportunities per denominator.

### Outcomes

Among each cohort detailed in the preceding subsection, we observed whether opportunities resulted in the patient having the corresponding screening test of interest, respectively, defined as at least 1 claim for a repeated DXA scan within 2 years of a prior DXA scan, an ECG within 30 days after an AHE, a Pap test within 7 days after an AHE, or a CXR within 7 days after an AHE (eTable 1 in the Supplement).^7,14,15^ Only tests for which claims could be definitively linked to the physician (via his or her personal encrypted identifier) attributed to the preceding opportunity were counted in the numerator.^15^ The expressed linkage of opportunities and any subsequent tests to a specific primary care physician was necessary to ensure that resulting measures of individual performance only involved components of care reasonably within his or her control.^28^

### Physician and Group Characteristics

For all physicians identified, we denoted their sex, international medical graduate status, and years since medical school graduation.^7,14,15^ We also investigated if they belonged to a billing group, defined as a group of 3 or more physicians who submit joint billings to the Ontario Ministry of Health and Long-Term Care. We recorded each billing group’s primary care model and size (number of physicians and number of rostered patients).^14,15^ Primary care models in Ontario differ with respect to their remuneration methods.^29,30^ For example, the primary mechanism for physician reimbursement is capitation in family health organizations, teams, and networks vs fee-for-service in family health groups.^29,30^

### Statistical Analysis

#### Ordering Frequency and Variation per Test

Individual physician and billing group ordering rates were independently calculated per test as the ratio of the number of tests ordered to the number of opportunities to order that test (eTable 1 in the Supplement). Rates for a given test were not calculated for physicians with fewer than 50 opportunities to order that test. This was done to avoid assessing individual performance on a minimum sample size, which might inaccurately reflect actual behavior and skew the overall distribution of use among physicians.^26^

To enable observation of ordering patterns across all 4 tests within a common physician cohort, we excluded any physicians with fewer than 50 opportunities to order any of the 4 tests from our analyses.^26,28,31^ To assess how this exclusion may have limited the generalizability of our results, baseline characteristics were compared by the number of tests for which a physician met the defined threshold, with analysis of categorical and continuous variables via χ^2^ tests of independence and Kruskal-Wallis tests, respectively. Within our final cohort, within-test variation was summarized among physicians and billing groups via interquartile ranges (IQRs).

#### Comparing Ordering Rates Among Tests

We stratified physician-level ordering rates into quintiles for each of the 4 low-value screening tests. Per physician, we assigned 1 point for each test for which he or she belonged to the top ordering quintile to denote use of that test as frequent. The total number of points (range, 0-4) was investigated to compute a physician’s cross-test score, a measure meant to assess physician tendency to frequently order low-value screening tests across all 4 scenarios.^32,33^ For example, a physician in the top ordering quintile for 3 of 4 tests would receive a score of 3. Physicians were classified according to their cross-test score as infrequent users (score, 0), isolated frequent users (score, 1), or generalized frequent users of 2 tests (score, 2), 3 tests (score, 3), or all tests (score, 4). The distribution of baseline characteristics by cross-test score was summarized and compared via χ^2^ tests of independence for categorical variables and via Kruskal-Wallis tests for continuous variables. To investigate whether physician-level ordering rates for the 4 tests were correlated, we used a 1-way χ^2^ test to compare observed and expected proportions (specified assuming independence of test rates) by cross-test score value.

#### Characteristics Associated With Generalized Frequent Users of Low-Value Screening

Physicians were dichotomized into infrequent or isolated frequent users (score, 0 or 1, respectively) vs generalized frequent users for 2 or more tests (score, ≥2). The cutoff value of 2 was informed by the median of the cross-test score distribution and the results of our 1-way χ^2^ tests indicating that physicians with frequent use of at least 1 test are more likely to frequently use another test. Furthermore, ordinal and binary versions of the cross-test score were found to have strong, positive correlation (ρ = 0.72, *P* < .001).

Multivariable mixed-effects logistic regression was used to assess a physician’s odds of generalized frequent use across tests, with adjustment for physician sex, international medical graduate status, and years since medical school graduation, in addition to the billing group characteristics (primary care model and the number of patients per physician).^14,15^ Random, group-specific intercepts were included to account for the clustered nature of the data (physicians within physician groups) and to enable calculation of 2 measures of group-level heterogeneity, the intracluster correlation coefficient and the median odds ratio.^34,35,36,37,38^ For mixed-effects logistic regression, any fixed-effect odds ratio is group specific.^34^ This is problematic for interpreting the association of a group characteristic, which has a constant value for all physicians within a group.^34^ Accordingly, we calculated interval odds ratios for group-level characteristics, which represents the middle 80% of the distribution of odds ratios comparing 2 physicians in 2 different groups with differing values of that characteristic (but identical values for all other group-level and physician-level covariates).^34,35,36,37^ Physicians with incomplete or missing data for any of the listed covariates were excluded, under the assumption that data were missing completely at random.

To investigate how the decision to combine infrequent users and isolated frequent users into a single category (score, <2) might have influenced the results of our primary regression analysis, we conducted a sensitivity analysis examining characteristics of physicians in the infrequent, isolated frequent, or generalized frequent user groups. Infrequent users were set as the reference group.

All analyses were performed using statistical software (SAS, version 9.4; SAS Institute Inc). Statistical significance was assessed using a 2-tailed *P* value of .05.

## Results

### Cohort Creation

The final sample consisted of 2394 primary care physicians (mean [SD] age, 51.3 [10.0] years; 50.2% female), who were predominantly Canadian medical school graduates (1701 [71.1%]), far removed from medical school graduation (median, 25.3 years; IQR, 17.3-32.3 years), and primarily reimbursed via fee-for-service in a family health group (1130 [47.2%]). Table 1 summarizes the assembly of all 4 test-specific cohorts between April 1, 2012, and March 31, 2016. Less than 1% of all opportunities initially identified per cohort were excluded because of incomplete or missing patient sociodemographic data. Before excluding physicians based on volume of opportunities, a total of 6 554 684 opportunities were identified among 11 448 primary care physicians. After excluding those without 50 or more opportunities for any test, we arrived at 8685 physicians (75.9%), who collectively had 6 255 414 opportunities. Of these physicians, 2394 physicians (27.6%) had 50 or more opportunities for all 4 tests, and these physicians ordered 59.6% of low-value tests observed. Overall, ECGs were the most commonly ordered test.

**Table 1.** Cohort Creation, Beginning With Screening Test–Specific Cohorts, Leading Into a Common Sample of Physicians

Variable	Screening Test–Specific Cohort, No.			
	Repeated DXA	ECG	Pap Test	CXR
Initial opportunities identified	818 328	5 226 436	5 226 436	5 226 436
Opportunities excluded^a^	−116 967	−2 140 101	−4 739 213	−2 946 691
Non-Ontario, Canada, resident	−1010	−7852	−7852	−7852
Missing age, sex, or postal code	−2352	−14 195	−14 195	−14 195
Male patients	NA	NA	−2 134 903	NA
Age not appropriate	−19 620	−111 028	−2 530 872	−111 028
Long-term care home residents	NA	−6142	NA	−6142
High-risk patient (not otherwise captured)^b^	NA	−2 000 884	−51 391	−2 807 474
Missing physician identifier^c^	−93 985	NA	NA	NA
Eligible opportunities	701 361	3 086 335	487 223	2 279 745
Opportunities excluded^a^	−377 832	−1 462 741	−190 006	−1 095 528
With physician with <50 opportunities to order test	−76 386	−49 753	−114 742	−58 369
With physician with <50 opportunities to order any test	−301 446	−1 412 988	−75 264	−1 037 159
Opportunities	323 529	1 623 594	297 217	1 184 217
Opportunities resulting in the corresponding test, No. (%)^d^	74 167 (22.9)	179 855 (11.1)	19 906 (6.7)	28 581 (2.4)

Abbreviations: CXR, chest radiograph; DXA, dual-energy x-ray absorptiometry; ECG, electrocardiogram; NA, not applicable; Pap, Papanicolaou.

^a^ Exclusions are denoted as negative values.

^b^ For the ECG cohort, this includes patients who are at high risk for cardiovascular disease; for the Pap cohort, this includes any patients with prior gynecologic cancer, prior hysterectomy, pregnancy in the past 9 months, or prior HIV infection; for the CXR cohort, this includes patients at high risk for cardiovascular or pulmonary disease. eTable 1 in the Supplement provides more details.

^c^ Only possible for repeated DXA measure because the initial annual health examination for the other 3 cohorts was not included unless an identifier for the physician who conducted the annual health examination was recorded on the corresponding claim.

^d^ Calculated as the proportion of opportunities resulting in the corresponding test divided by opportunities.

eTable 2 in the Supplement compares baseline characteristics by the number of tests for which a physician met the minimum number of opportunities. Physicians who did not meet the minimum threshold for all 4 tests had fewer median years in practice and a lower median number of patients per physician than those who did.

### Test Ordering Frequency and Variation

Among our cohort of 2394 physicians, the median physician-level ordering rates were 19.5% (IQR, 13.3%-27.6%) for repeated DXA scans, 4.8% (IQR, 1.9%-14.3%) for ECGs, 5.1% (IQR, 1.9%-10.3%) for Pap tests, and 0.9% (IQR, 0.4%-1.9%) for CXRs (Table 2). Among 540 billing groups, the median billing group–level ordering rates were 19.7% (IQR, 14.5%-25.6%) for repeated DXA scans, 6.1% (IQR, 2.9%-12.8%) for ECGs, 7.3% (IQR, 4.7%-11.1%) for Pap tests, and 1.2% (IQR, 0.7%-2.0%) for CXRs.

**Table 2.** Descriptive Statistics for Physician-Level Ordering Rates by Screening Test

Variable	Overall	Top 20%	Bottom 80%
**Repeated DXA**			
No.	2394	479	1915
Range, %	0.0-69.0	29.8-69.0	0.0-29.7
Mean (SD), %	21.2 (10.9)	38.0 (6.9)	17.0 (7.0)
Median (IQR), %	19.5 (13.3-27.6)	36.0 (32.6-42.2)	17.1 (11.9-22.6)
CV	1.95	5.54	2.45
**ECG**			
No.	2394	478	1916
Range, %	0.0-94.4	17.7-94.4	0.0-17.7
Mean (SD), %	11.0 (14.6)	35.5 (15.3)	4.9 (4.5)
Median (IQR), %	4.8 (1.9-14.3)	31.3 (23.5-44.5)	3.3 (1.5-7.2)
CV	0.76	2.33	1.11
**Pap Test**			
No.	2394	478	1916
Range, %	0.0-84.0	12.5-84.0	0.0-12.4
Mean (SD), %	8.0 (9.4)	22.6 (11.5)	4.3 (3.4)
Median (IQR), %	5.1 (1.9-10.3)	18.8 (14.8-25.9)	3.7 (1.4-6.8)
CV	0.85	1.97	1.27
**CXR**			
No.	2394	479	1915
Range, %	0.0-70.6	2.4-70.6	0.0-2.4
Mean (SD), %	2.2 (4.8)	7.5 (8.9)	0.8 (0.6)
Median (IQR), %	0.9 (0.4-1.9)	4.3 (3.0-7.3)	0.7 (0.4-1.2)
CV	0.45	0.84	1.34

Abbreviations: CV, coefficient of variation; CXR, chest radiograph; DXA, dual-energy x-ray absorptiometry; ECG, electrocardiogram; IQR, interquartile range; Pap, Papanicolaou.

The eFigure in the Supplement shows the frequency distributions of physician ordering rates per test descriptive statistics both by and irrespective of ordering quintile per test. The top ordering quintile cutoff values were 29.8% for repeated DXA scans, 17.7% for ECGs, 12.4% for Pap tests, and 2.4% for CXRs. Physician-level ordering variability was most pronounced for repeated DXA scans (IQR, 13.3%-27.6%) and least pronounced for CXRs (IQR, 0.4%-1.9%).

### Physician Ordering of Multiple Low-Value Tests

The Figure shows the percentage frequency of physicians (n = 2394) and tests (n = 302 509) by cross-test score. Of total tests ordered, 74 167 were DXA scans; 179 855, ECGs; 19 906, Pap tests, and 28 581 CXRs. Physicians who were male (odds ratio, 1.29; 95% CI, 1.01-1.64), further removed from medical school graduation (odds ratio, 1.03; 95% CI, 1.02-1.04), or in an enhanced fee-for-service payment model (family health group) vs a capitated payment model (family health team) (odds ratio, 2.04; 95% CI, 1.42-2.94) had increased odds of being generalized frequent users. Overall, 18.4% (441 of 2394) of physicians were classified as generalized frequent users and were responsible for ordering 39.2% (118 665 of 302 509) of all low-value tests. The results of our 1-way χ^2^ test for the cross-test score indicate that observed proportions of physicians across the 5 possible levels significantly deviate from expected proportions, assuming independence of the different testing rates (χ^2^_4_ = 22.3, *P* < .001). The significant result appears to be driven by a greater proportion of physicians observed with frequent use of ECGs and CXRs (7.8%, or 186 of 2394 physicians) than expected (4.0%, or 95 of 2394 physicians) if ordering rates were truly independent (eTable 3 in the Supplement).

**Figure.**
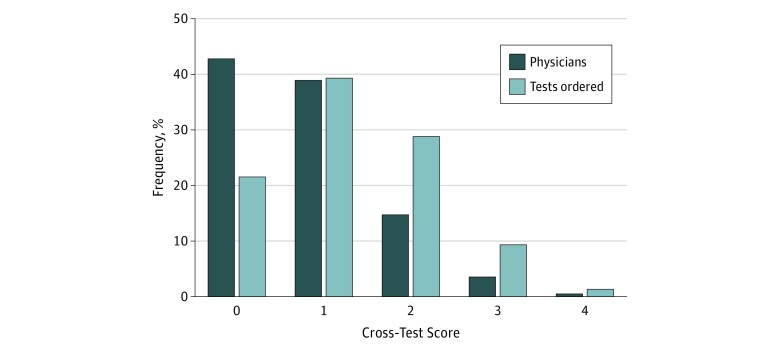
Percentage Distribution of Physicians and Potentially Low-Value Tests Ordered Grouped by Cross-Test Score Data are shown for 2394 primary care physicians and for 302 509 low-value screening tests.

### Characteristics Associated With Generalized Frequent Use of Low-Value Screening

Table 3 summarizes physician-level and group-level characteristics by cross-test score. In general, physicians who were male, further removed from medical school graduation, and members of a billing group with a lower volume of patients per physician and a payment model based on fee-for-service (ie, family health group) were more likely to have a higher cross-test score.

**Table 3.** Physician-Level and Group-Level Characteristics by Cross-Test Score Among 2394 Physicians

Variable	Cross-Test Score, No. (%)					*P* Value^a^
	0	1	2	3	4	
Physicians (n = 2394)	1023 (42.7)	930 (38.8)	349 (14.6)	82 (3.4)	10 (0.4)	NA
Total screening tests ordered (n = 302 509)	65 162 (21.5)	118 682 (39.2)	86 963 (28.7)	28 079 (9.3)	3623 (1.2)	NA
Ordering opportunities^b^ (n = 3 428 557)	1 469 106 (42.8)	1 330 405 (38.8)	498 338 (14.5)	118 006 (3.4)	12 702 (0.4)	NA
Any test rate, %^c^	4.4	8.9	17.5	23.8	28.5	NA
**Physician Level**						
Sex						
Male	467 (45.7)	468 (50.3)	201 (57.6)	52 (63.4)	<6 (NA)^d^	<.001^e^
Female	556 (54.3)	462 (49.7)	148 (42.4)	30 (36.6)	<6 (NA)^d^	
IMG						
Yes	295 (28.8)	286 (30.8)	95 (27.2)	13 (15.9)	<6 (NA)^d^	.05
No	728 (71.2)	644 (69.2)	254 (72.8)	69 (84.1)	<6 (NA)^d^	
Years since medical school graduation, median (IQR)	23.3 (16.3-30.3)	25.3 (18.3-32.3)	27.3 (19.3-36.3)	27.8 (19.3-36.3)	30.8 (22.3-49.3)	<.001^e^
**Group Level**						
Primary care model^f^						
Family health group	436 (42.6)	450 (48.4)	187 (53.6)	49 (59.8)	8 (80.0)	<.001^e^
Family health organization	295 (28.8)	253 (27.2)	101 (28.9)	26 (31.7)	<6 (NA)^d^	
Family health team	288 (28.2)	220-230 (NA)^d^	61 (17.5)	7 (8.5)	<6 (NA)^d^	
Other^g^	6 (0.4)	<10 (NA)^d^	0	0	0	
No. of patients per physician, median (IQR)	90.0 (37.2-177.1)	84.0 (36.9-177.6)	85.1 (31.8-187.8)	75.5 (20.7-128.7)	25.7 (22.7-30.7)	.04^e^
Missing	29 (2.8)	13 (1.4)	10 (2.9)	<6 (NA)^d^	<6 (NA)^d^	NA

Abbreviations: IMG, international medical graduate; IQR, interquartile range; NA, not applicable.

^a^ *P* values for continuous variables produced via Kruskal-Wallis tests comparing the distribution of baseline characteristics by the number of tests in the top ordering quintile. *P* values for categorical variables produced from χ^2^ tests of independence (or Fisher exact test if ≥1 cell[s] with an expected count of 0 or if ≥25% of expected cell counts <5).

^b^ Total number of annual health examinations and repeated dual-energy x-ray absorptiometry scans attributed to a physician, collectively referred to as opportunities for test ordering.

^c^ Calculated as total screening tests ordered divided by ordering opportunities.

^d^ Handled small cell counts and the corresponding proportions according to *ICES’ Working With Small Cells Guidelines*^21^ (ie, small cell frequencies of <6 suppressed as <6 with proportion as NA). If suppression was not enough to prevent calculation of true value in a suppressed cell via back subtraction, we reported the range of values for another cell in the same column for that same variable.

^e^ Significant at *P* < .05.

^f^ Represents the primary care patient enrollment model that informs group organization and remuneration.

^g^ Other primary care models (including family health networks) were collapsed into “other” category because of low observed frequency.

Table 4 summarizes our mixed-effects logistic regression results. Physicians who were male, domestic medical graduates, or with more years since medical school graduation had significantly greater odds of being generalized frequent users. Physicians in a family health team (a type of capitated payment model) were less likely to be generalized frequent users than peers at a family health group based on fee-for-service. The 1.62 median odds ratio exceeds all odds ratios estimated by the model, suggesting that the unexplained heterogeneity between billing groups is of greater relevance than any measured patient-level or physician-level characteristic for understanding an individual physician’s odds of generalized frequent use.

**Table 4.** Physician-Level and Group-Level Characteristics Associated With Generalized Frequent Use of 2 or More Screening Tests (ie, Cross-Test Score ≥2) Among 2340 Physicians^a^

Variable	Odds Ratio (95% CI)^b^	80% Interval Odds Ratio^b^^,^^c^
**Physician-Level Fixed Effects**		
Male vs female	1.29 (1.01-1.64)^d^	NA
IMG vs domestic	0.64 (0.49-0.84)^d^	NA
Years since medical school graduation	1.03 (1.02-1.04)^d^	NA
**Group-Level Fixed Effects**		
Primary care model^e^		
Family health organization vs family health group	0.90 (0.66-1.23)	(0.44-1.85)
Family health team vs family health group	0.49 (0.34-0.71)^d^	(0.23-1.07)
No. of patients per physician		
<30-60 vs <0	0.67 (0.43-1.03)	(0.28-1.57)
>60 to 110 vs <30	0.91 (0.60-1.38)	(0.39-2.10)
>110 to 210 vs <30	0.69 (0.44-1.07)	(0.29-1.62)
>210 vs <30	0.81 (0.53-1.26)	(0.35-1.91)
**Group-Level Random Effects**^b^		
Variance (SE)	0.26 (0.12)	NA
Median odds ratio^c^	1.62 (1.39-1.86)	NA
Intracluster correlation coefficient, %^c^^,^^f^	7.27	NA

Abbreviations: IMG, international medical graduate; NA, not applicable.

^a^ All reported values are based on SAS PROC GLIMMIX output; model estimation method is Laplace; denominator *df* estimation method is between and within; covariance structure is standard variance.

^b^ Adjusted for all other factors present in the model and table, as well as the quarter and season in which the visit occurred.

^c^ Estimated using group-level variance estimate.

^d^ Significant at *P *< .05.

^e^ Represents the primary care patient enrollment model that informs group organization and remuneration. Suppressed comparison of other vs family health group because of low observed frequency of the other category.

^f^ Calculated using the latent response formulation (ie, set residual variance equal to 3.29).

The results of our sensitivity analysis are summarized in eTable 4 in the Supplement. No measured characteristics were significantly associated with physicians’ odds of infrequent use vs isolated frequent use. Furthermore, the results of the logit modeling physicians’ odds of generalized frequent use vs infrequent use concur with those from our primary regression model.

## Discussion

In this large, retrospective cohort study of primary care physicians in Ontario, we observed frequent ordering of low-value screening tests, with widespread physician-level variation.^22,23,24,25^ Within our cohort, 18.4% (441 of 2394) of physicians were classified as generalized frequent users (ie, in the top 20% by ordering rate for ≥2 low-value screening tests) and ordered 39.2% of all low-value tests. Generalized frequent users were more likely to be male, trained domestically, with more years in practice, and part of a billing group primarily remunerated via fee-for-service. The results demonstrate that, while most physicians exhibit infrequent or isolated frequent use of low-value screening tests, there is a minority of physicians who are responsible for a large proportion of all low-value testing ordered. These results have potential implications for the future design of interventions to reduce low-value testing.

Our findings show that a subgroup of primary care physicians demonstrating a pattern of overuse across multiple screening scenarios ordered a disproportionate number of low-value tests. While this finding is novel, review of prior literature suggests that this result is not surprising. First, prior work has clearly demonstrated significant ordering variation in low-value testing among individual physicians, with positive correlations between ordering rates of different low-value tests.^7,14,15,39,40^ For example, a prior study by Makarov and colleagues^40^ found regional associations between low-value breast and prostate cancer imaging, suggesting that regional physician practice explains some of the ordering variability in low-value imaging tests. Furthermore, a study by Lipitz-Snyderman and colleagues^41^ demonstrated that use of low-value cancer care was physician dependent, rather than driven by patient needs or preferences. Our study builds on these results by describing, for the first time to date, a small group of individual physicians who order a high frequency of these low-value screening tests, which represent a significant portion of the total low-value care ordered.

Consistent with prior research, our study found that physicians who were male and further removed from medical school graduation were more likely to frequently order low-value care.^14,15^ Physicians in fee-for-service payment models were also more likely to order a higher frequency of low-value care than those in capitated models. These findings fit into a previously described framework of the drivers of low-value care, which includes factors at both the professional and health system level.^42^ Even after adjustment for many physician-level and group-level characteristics, we found a substantial degree of unexplained variation, suggesting other potential, unmeasured drivers of physician ordering. Understanding how these drivers, including health system drivers (eg, physician payment models), influence physician ordering behavior will be critical in the design of future interventions.

Worldwide CW campaigns have aimed to raise general awareness among physicians and patients on the issue of low-value care. The importance of reducing low-value care is not only to avoid the cost of the initial screening test but also to prevent unnecessary downstream care resulting from false-positive results.^14^ Local interventions designed to reduce low-value care, such as computerized decision support tools or audit and feedback, are usually implemented for all physicians.^3^ To date, the results of CW campaigns have been marginal, possibly because the interventions are either not intensive enough to influence change or are too broadly implemented.^4,9,43,44^ Most physicians do not order low-value care frequently enough for interventions to make a significant difference in ordering practice.^9,14,15^ Worse, the increased administrative burden that goes along with most interventions, such as decision support tools, may lead to increased frustration and physician burnout. Our data suggest that future interventions should consider a more focused, intensive approach on the minority of physicians who frequently order low-value care. One could imagine a higher-intensity intervention aimed at the minority of high-ordering physicians (eg, academic detailing) and a lower-intensity intervention (eg, broad awareness and education campaigns) aimed at physicians who order low-value care infrequently. Such an approach would avoid exposing physicians who infrequently order low-value care to potentially burdensome quality improvement initiatives for which the marginal benefit may be limited and may be more cost-effective.^33,45^ More research on the effectiveness of such a multipronged strategy to reduce low-value care is warranted.

### Limitations

Our findings should be considered within the context of the study’s limitations. First, administrative data lack clinical granularity, such as new symptoms, physical examination findings, and laboratory data, which may inform the reason for the test being ordered.^8^ The physicians in our cohort were more likely to be further removed from medical school graduation and have a higher median number of patients per physician than those physicians who were ultimately excluded. These systematic differences could limit the generalizability of the results, particularly given that years in practice was significantly associated with increased odds of being a generalized frequent user. In addition, our investigation of a test’s value did not account for considerations beyond the scope of CW recommendations.^22,23,24,25^ For instance, if advocating against the provision of these 4 low-value tests causes physicians to use more expensive or invasive alternatives for low-risk patients, then the overall influence of these 4 tests is not as negative as CW recommendations would suggest.^14,39^ Our study only examined 4 tests,^7,14,15^ and investigating low-value care ordering patterns across a greater number of tests, treatments, and procedures may better describe the extent to which low-value care provision is correlated across services and help create more reliable physician profiles.^28,32^

## Conclusions

This large, retrospective population-based cohort study identified a group of physicians who ordered a high frequency of low-value care that was responsible for almost 40% of all low-value tests ordered. Future investigations are warranted to focus on understanding drivers of low-value care and interventions to reduce its frequency, particularly in the generalized frequent user physician population.

## Notes

Supplement. **eTable 1.** Cohort Creation (Denominator) and Outcome Definitions (Numerator) per Recommendation **eTable 2.** Baseline Characteristics by Number of Tests With 50+ Opportunities (n = 11,448) **eTable 3.** Observed and Expected Frequency Distribution of Physicians Based on Cross-Test Score and Frequently Used Test(s) **eTable 4.** Results of Mixed-Effects Multinomial Logistic Regression (n = 2,340) **eFigure.** Frequency Distributions for Unadjusted Physician-Level Ordering Rates for Repeated DXA Scans, ECGs, Pap Tests, and CXRs
