# Supplementary material for: Physician Characteristics Associated With Ordering 4 Low-Value Screening Tests in Primary Care
Source: JAMA Netw Open. 2018 Oct 12;1(6):e183506. doi: 10.1001/jamanetworkopen.2018.3506 (PMC6324437; doi:10.1001/jamanetworkopen.2018.3506)
Supplement: Supplement. — eTable 1. Cohort Creation (Denominator) and Outcome Definitions (Numerator) per Recommendation eTable 2. Baseline Characteristics by Number of Tests With 50+ Opportunities (n = 11,448) eTable 3. Observed and Expected Frequency Distribution of Physicians Based on Cross-Test Score and Frequently Used Test(s) eTable 4. Results of Mixed-Effects Multinomial Logistic Regression (n = 2,340) eFigure. Frequency Distributions for Unadjusted Physician-Level Ordering Rates for Repeated DXA Scans, ECGs, Pap Tests, and CXRs [file jamanetwopen-1-e183506-s001.pdf]

Supplementary Online Content

Bouck Z, Ferguson J, Ivers NM, et al. Physician characteristics associated with ordering 4 low-value screening tests in primary care. *JAMA Netw Open*. 2018:1(6):e183506. doi:10.1001/jamanetworkopen.2018.3506

eTable 1. Cohort Creation (Denominator) and Outcome Definitions (Numerator) per Recommendation

eTable 2. Baseline Characteristics by Number of Tests With 50+ Opportunities (n = 11,448)

eTable 3. Observed and Expected Frequency Distribution of Physicians Based on Cross-Test Score and Frequently Used Test(s)

eTable 4. Results of Mixed-Effects Multinomial Logistic Regression (n = 2,340)

eFigure. Frequency Distributions for Unadjusted Physician-Level Ordering Rates for Repeat DXA Scans, ECGs, Pap Tests, and CXRs

This supplementary material has been provided by the authors to give readers additional information about their work.

© 2018 Bouck Z et al. *JAMA Network Open*.

eTable 1. Cohort Creation (Denominator) and Outcome Definitions (Numerator) per Recommendation

Recommendation	Denominator	Numerator
Don't repeat DXA scans more often than every two years in the absence of high-risk or new risk factors[23,24]	*Inclusion criteria:* Prior baseline, low-risk, or high-risk DXA scan[7].<br><br>*Exclusion criteria:* Age <40 or >105 yr on date of scan[7].	Patients meet inclusion and exclusion criteria with claim for subsequent DXA scan within 2 years of prior scan[7].
Don't ordering screening electrocardiograms (ECGs) for asymptomatic or low-risk outpatients[21,22]	*Inclusion criteria:* Periodic health visit with a primary care physician[14].<br><br>*Exclusion criteria:* Prior diagnosis or recent symptoms suggestive of cardiovascular disease; prior cardiac procedures; high-risk comorbidities; age <18 or >105 yr[14].	Patients meet inclusion and exclusion criteria with receipt of an ECG within 30 days of periodic health visit[14].<br><br>Exclude any ECGs that occur during an emergency department visit from the numerator only[b].
Don't order routine chest X-rays (CXRs) for asymptomatic or low-risk outpatients[22]	*Inclusion criteria:* Periodic health visit with a primary care physician[15].<br><br>*Exclusion criteria:* Prior diagnosis or recent symptoms suggestive of cardiopulmonary disease; prior cardiac procedures; consultations with cardiopulmonary specialists; high-risk comorbidities; age <18 or >105 yr[15].	Patients meet inclusion and exclusion criteria with claim for CXR within 7 days of periodic health visit[15].<br><br>Exclude any CXRs done during inpatient stay or emergency department visit from numerator only[15].
Don't screen women with Pap smears if under 21 years of age or over 69 years of age[22]	*Inclusion criteria:* Periodic health visit with a primary care physician[b].<br><br>*Exclusion criteria:* Previous gynecologic cancer diagnosis; previous hysterectomy; recent pregnancy; HIV infection age <13 or >105 yr; age ≥21 or ≤69; male sex[7].	Patient meets inclusion and exclusion criteria with claim for cervical cancer screening with Pap smear within 7 days of periodic health visit[7].

Notes: DXA = dual-energy X-ray absorptiometry; ECG = electrocardiogram; Pap = Papanicolaou; CXR = chest X-ray; HIV = human immunodeficiency virus.

[a] Both components of the same CW recommendation; however, split to reflect independent measurement, which is consistent with preceding studies.

[b] Represent methodological deviations from referenced studies.

© 2018 Bouck Z et al. *JAMA Network Open*.

eTable 2. Baseline Characteristics by Number of Tests With 50+ Opportunities (n = 11,448)

Characteristic	Number of tests with 50+ opportunities			P value[a]
	0	1-3	4	
	N=2,763	N=6,291	N=2,394	
Age, median (IQR)	46 (34-60)	49 (38-58)	51 (44-58)	<.001[b]
Missing (n, %)	310 (11.2)	282 (4.5%)	0 (0.0%)	
Years since graduation, median (IQR)	16 (4-32)	22 (9-31)	25 (17-32)	<.001[b]
Missing (n, %)	43 (1.6)	16 (0.2)	0 (0.0)	
Sex, n (%)				<.001
Male	1,500 (54.3)	3,412 (54.2)	1,193 (49.8)	
Female	1,225 (44.3)	2,864 (45.5)	1,201 (50.2)	
Missing	38 (1.4)	15 (0.2)	0 (0.0)	
IMG, n (%)				<.001
Non-IMG	1,734 (62.8)	4,596 (73.1)	1,701 (71.1)	
IMG	338 (12.2)	1,329 (21.1)	693 (28.9)	
Missing	691 (25.0)	366 (5.8)	0 (0.0)	
Primary care model				<.001
Primarily fee-for-service	1,961 (71.0)	2,722 (43.3)	1,130 (47.2)	
Primarily capitation	754 (27.3)	3,502 (55.7)	1,258 (52.5)	
Other	48 (1.7)	67 (1.0)	6 (0.3)	
Patients per physician, median (IQR)	24 (3-84)	70 (28-152)	85 (36-178)	<.001[b]
Missing (n, %)	1,762 (63.8)	1,659 (26.4)	54 (2.3)	

Notes: *Significant at P<.05.
[a] Calculated from three-way tests.
[b] Missing values were excluded from the statistical test to which the P-value corresponds.

© 2018 Bouck Z et al. *JAMA Network Open*.

eTable 3. Observed and Expected Frequency Distribution of Physicians Based on Cross-Test Score and Frequently Used Test(s)

Cross-test score[b]	No. physicians			Frequently used test(s)[a]			
	Observed	Expected[c]	Relative deviation[d]	DEXA	ECG	CXR	Pap
0	1023 (42.73)	981 (40.96)	0.043	O	O	O	O
1	276 (11.53)	245 (10.24)	0.126	X	O	O	O
1	180 (7.52)	245 (10.24)	-0.266	O	X	O	O
1	203 (8.48)	245 (10.24)	-0.172	O	O	X	O
1	271 (11.32)	245 (10.24)	0.105	O	O	O	X
2	46 (1.92)	61 (2.56)	-0.249	X	X	O	O
2	40 (1.67)	61 (2.56)	-0.347	X	O	X	O
2	53 (2.21)	61 (2.56)	-0.135	X	O	O	X
2	121 (5.05)	61 (2.56)	0.974	O	X	X	O
2	51 (2.13)	61 (2.56)	-0.168	O	X	O	X
2	38 (1.59)	61 (2.56)	-0.380	O	O	X	X
3	27 (1.13)	15 (0.64)	0.762	X	X	X	O
3	15 (0.63)	15 (0.64)	0.021	X	X	O	X
3	12 (0.50)	15 (0.64)	-0.217	X	O	X	X
3	28 (1.17)	15 (0.64)	0.827	O	X	X	X
4	10 (0.42)	4 (0.16)	1.611	X	X	X	X

Notes: [a] X and O respectively indicate whether a physician in that row belongs to or does not belong to the top ordering quintile for that test.

[b] Represents number of tests in the top ordering rate quintile.

[c] Minor discrepancy in observed and expected column totals due to rounding to closest integer per cell.

[d] Calculated as (% observed - % expected)/(% expected).

© 2018 Bouck Z et al. *JAMA Network Open*.

eTable 4. Results of Mixed-Effects Multinomial Logistic Regression (n = 2,340)

Fixed Effects		Cross-test score	OR[a] (95% CI)	IOR-80[a,b]
Physician-level				
Sex, male v female		1 v 0	1.08 (0.89-1.31)	-
		≥2 v 0	1.33 (1.03-1.73)*	-
IMG v non-IMG		1 v 0	1.04 (0.84-1.28)	-
		≥2 v 0	0.65 (0.49-0.87)*	-
Years since graduation		1 v 0	1.00 (1.01-1.02)	-
		≥2 v 0	1.04 (1.03-1.05)*	-
Group-level				
Primary care model[c, d]				
	FHO v FHG	1 v 0	0.87 (0.68-1.11)	(0.45, 1.65)
		≥2 v 0	0.83 (0.60-1.17)	(0.39, 1.76)
	FHT v FHG	1 v 0	0.81 (0.63-1.04)	(0.42, 1.55)
		≥2 v 0	0.44 (0.30-0.65)*	(0.20, 0.99)
No. patients per physician				
	30-59 v <30	1 v 0	1.14 (0.82-1.59)	(0.54, 2.40)
		≥2 v 0	0.71 (0.45-1.13)	(0.29, 1.71)
	60-109 v <30	1 v 0	1.03 (0.75-1.42)	(0.50, 2.15)
		≥2 v 0	0.91 (0.59-1.42)	(0.39, 2.16)
	110-209 v <30	1 v 0	0.95 (0.69-1.32)	(0.45, 2.00)
		≥2 v 0	0.67 (0.42-1.06)	(0.27, 1.61)
	>210 vs <30	1 v 0	1.08 (0.78-1.50)	(0.51, 2.26)
		≥2 v 0	0.84 (0.53-1.33)	(0.35, 2.01)
Random Effects[a]				
Group-level				
Variance (SE)		1 v 0	0.04 (0.05)	
		≥2 v 0	0.25 (0.12)	
MOR[b]		1 v 0	1.21 (1.11-1.30)*	
		≥2 v 0	1.62 (1.38-1.85)*	
ICC, %[b,e]		1 v 0	1.16	
		≥2 v 0	7.15	

Notes: Significant at $P \leq .05$*. OR = odds ratio; CI = confidence interval; IOR-80 = 80% interval odds ratio; IMG = international medical graduate; FHO = family health organization; FHG = family health group; FHT = family health team; MOR = median odds ratio. All reported values based on SAS PROC GLIMMIX output; model estimation method = Laplace; denominator degrees of freedom estimation method = between and within (bw); covariance structure = standard variance (vc).

[a] Adjusted for all other factors present in the model/table, as well as the quarter and season in which visit occurred.

[b] Estimated using group-level variance estimate.

[c] Represents the primary care patient enrollment model which informs group organization and remuneration.

[d] Suppressed comparison of 'Other v FHG' due to low observed frequency of 'Other' category.

[e] Calculated using the latent response formulation (i.e. let residual variance = 3.29).

© 2018 Bouck Z et al. *JAMA Network Open*.

eFigure. Frequency Distributions for Unadjusted Physician-Level Ordering Rates for Repeat DXA Scans, ECGs, Pap Tests, and CXRs *Notes:* DXA = dual-energy X-ray absorptiometry; ECG = electrocardiogram; Pap = Papanicolaou; CXR = chest X-ray.

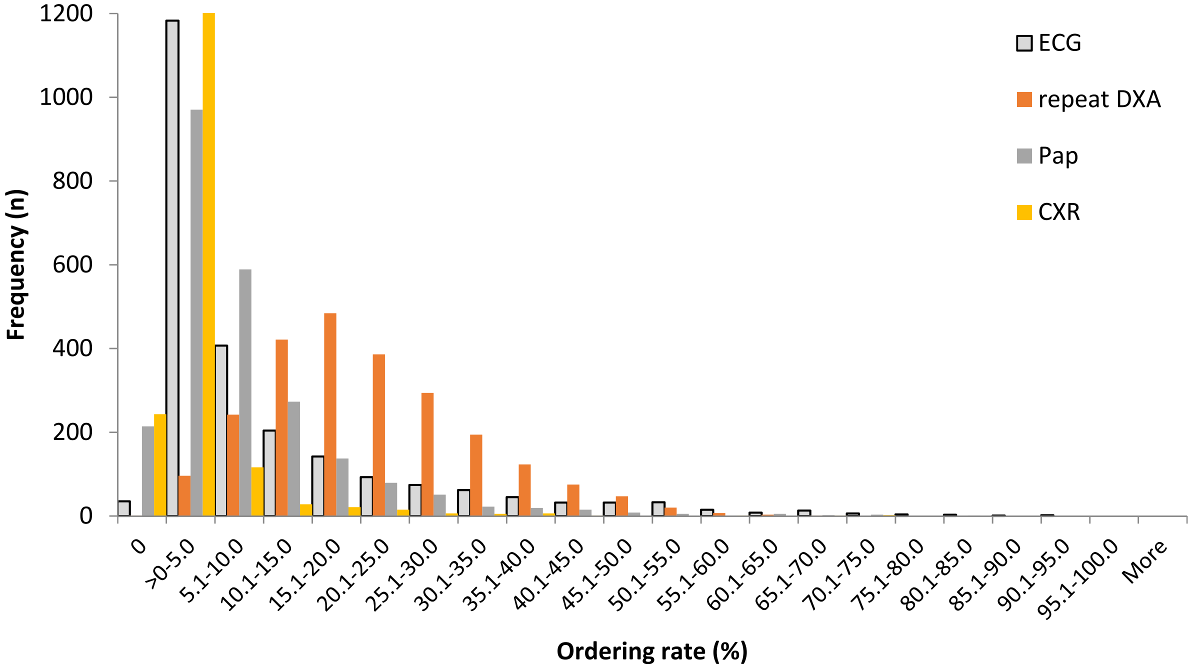

© 2018 Bouck Z et al. *JAMA Network Open*.
